# Suppressing HOXA-10 Gene Expression by MicroRNA 135b During the Window of Implantation in Infertile Women

**Published:** 2020

**Authors:** Aida Riyanti, Ririn Rahmala Febri, Sarah Chairani Zakirah, Achmad Kemal Harzif, Rajuddin Rajuddin, Raden Muharam, Asmarinah Asmarinah, Budi Wiweko

**Affiliations:** 1- Division of Reproductive Endocrinology and Infertility, Department of Obstetrics and Gynecology, Faculty of Medicine, Universitas Indonesia, Jakarta, Indonesia; 2- Human Reproductive, Infertility, and Family Planning Research Center, Indonesian Medical Education and Research Institute (IMERI), Faculty of Medicine, Universitas Indonesia, Jakarta, Indonesia; 3- Yasmin IVF Clinic, Dr. Cipto Mangunkusumo General Hospital, Jakarta, Indonesia; 4- Department of Obstetrics and Gynecology, Faculty of Medicine, Universitas Syiah Kuala, Banda Aceh, Indonesia; 5- Department of Medical Biology, Faculty of Medicine, Universitas Indonesia, Jakarta, Indonesia

**Keywords:** Endometrium, HOXA-10, Infertility, miRNA 135b, Window of implantation

## Abstract

**Background:**

Implantation failure has long been identified as a common problem underlying low success rate of IVF. Currently, endometrial receptivity has gained expert attention as it is demonstrated to contribute to successful embryo implantation. MicroRNAs (miRNAs) is known to affect endometrial receptivity through post-transcriptional gene expression regulation. This study aimed to evaluate the expression of miRNA 135b and HOXA-10 during the implantation window in endometrial tissue of infertile women.

**Methods:**

A total of 14 patients diagnosed with infertility in the gynaecology clinic of Cipto Mangunkusumo and Daya Medika hospitals Jakart, Indonesia were selected as the observed group, and 9 fertile patients were enrolled in the control group. Total RNA was isolated from endometrial tissues collected at the secretory phase of the menstrual cycle. The miRNA 135b and HOXA-10 mRNA expression were measured using quantitative real-time PCR (qPCR). The correlation between these variables was then determined using Pearson’s correlation coefficient.

**Results:**

The expression of miRNA 135b in the infertile group was significantly higher by 1.81-fold compared to the control group (p<0.01), whereas, expression of HOXA-10 mRNA was significantly lower in the infertile group compared to the controls (p=0.047). Significant negative correlation was observed between the expression of miRNA 135b and HOXA-10 mRNA in infertile women (p=0.021; r=−0.607).

**Conclusion:**

Taken together, this study provides that alteration of miRNA expression is involved in regulating the implantation process partly via modulation of the expression of gene required for implantation.

## Introduction

Infertility was defined as the inability to achieve pregnancy after 1 year of regular sexual intercourse without contraception, and it remains as a highly prevalent condition worldwide (1). Even though ART is known as the most efficacious therapy for infertility, the pregnancy rate of IVF-ET remains relatively low to date (2). In Indonesia, only approximately 29.05% of the embryos transferred into the uterus resulted in successful pregnancies. Several factors have been known to affect the success of implantation including embryo quality, the interaction between the endometrium and the embryo, and endometrial receptivity, of which insufficient endometrial receptivity is responsible for approximately 60% of the implantation failures (3).

Endometrial receptivity is a process which comprises many expressions of molecular complexes. During the window of implantation (WOI), within the day 19 to 24 of a regular menstrual cycle, the endometrium enhances its adhesion capability to improve the implantation process for embryo invasion (4). Moreover, the endometrial gene expression profile is modified under the influence of the ovarian hormones, namely estrogen and progesterone (5). Some studies also reported that these hormones regulate some factors involved in implantation, such as growth factor, cytokine, chemokine, lipid, and adhesion molecules (6,7).

Previous studies have reported that a number of gene expressions are associated with endometrial receptivity. The regulation of implantation involves several molecules; however, the mechanism remains poorly understood (8). One of the widely reported potential regulators of endometrial receptivity is microRNAs (miRNAs) (2). miRNAs are a family of small, non-coding RNA which regulate gene expression in a sequence-specific manner. They play a crucial role in some biological events including implantation, differentiation, development, proliferation, and signal transduction (9). Kuokkanen et al. reported that some gene expressions are decreased during the secretory phase of the epithelial endometrium cell cycle (10). It is believed that most miRNAs negatively regulate their target gene expression by causing degradation of mRNA transcripts or translational repression. Besides, abnormality of miRNA expression has been known to pertain to endometrial disease, and carcinoma.

MicroRNA is known as an important regulator of multiple signal transductions pathways, including the expression of HOXA-10. HOXA-10 protein functions as a transcription factor that controls the expression of other vital proteins such as cell adhesion molecules and metabolic mediators. Petracco et al. suggested that a surge in micro-RNA 135b expression during the secretory phase of the uterus correlates with a decrease in HOXA-10 expression, leading to aberrant endometrial receptivity in endometriosis patients (11).

Accordingly, the goal of this study was to substantiate the interdependent expression of micro-RNA 135b and HOXA-10 in the endometrium during the implantation window of infertile women.

## Methods

### Subjects and tissue samples:

This study was a cross-sectional one, which was conducted for 6 months from October 2018 to April 2019 at Dr. Cipto Mangunkusumo General Hospital and Daya Medika IVF Clinic Jakarta, Indonesia. Twenty three women with ages ranging from 20–40 years old were involved, which were categorized into 2 groups; 9 subjects were in the control group and 14 subjects in the observed infertile group. All patients included in the study gave their written informed consents. The inclusion criteria of control subjects were women who had at least 1 normal pregnancy and delivery and had attended the clinic to undergo pap-smear and screening. Moreover, the inclusion criteria for the infertile group were women undergoing IVF procedure due to infertility conditions who had gone through various standard diagnoses to evaluate their hormones, partners’ sperm conditions and ovarian functions. Additionally, women in the infertile group had mid-secretory endometrium thickness of ≥8 *mm*, regular menstruation cycle (28–31 days) and normal uterus sonography through USG and/or hysteroscopy.

Eutopic endometrium tissue was obtained from all women by an endometrial biopsy using a sterile Pipelle cannula (Pipelle de Cornier, France) within day 20–24 of the menstrual cycle. The biopsy process comprised two parts; the first part was to fix the endometrium tissues in 10% buffered formalin for histology to confirm the tissue collection at the secretory phase of the menstrual cycle, the second part was for the immediate storage of biopsy samples at −20°*C* for downstream processes and analysis.

### Human rights statements and informed consent:

All procedures followed were in accordance with the ethical standards of the responsible committee on human experimentation from the ethics committee of the Faculty of Medicine, Universitas Indonesia and with the Helsinki Declaration of 1964 and its later amendments. Informed consent was obtained from all patients and controls for being included in the study.

### Approval by ethics committee:

All procedures followed were approved by the ethical standards from ethics committee of Faculty of Medicine, Universitas Indonesia (No: 980/UN2.F1/ETHIC/VIII/2018).

### RNA isolation, cDNA synthesis, and qPCR:

RNA was isolated from biopsied endometrial tissues using the RNEasy Mini Kit (Qiagen, Germany) according to the manufacturer’s protocol. The RNA quantity and quality were assessed through NanoDrop-2000 spectrophotometry at 260 and 280 *nm* wavelengths. Synthesis of complementary DNA (cDNA) was performed using QuantiTect Reverse Transcription (Qiagen, Germany). Quantitative polymerase chain reaction (qPCR) was performed using QuantiFast SYBR Green PCR Kit (Qiagen, Germany) for HOXA10, using the absolute quantification. The amount of mRNA was quantified by titration of an unknown amount of target template against a dilution series of known amount of the standard. The primer used for HOXA10 mRNA expression analysis was forward 5′-CCTTCCGAGAGCAGCAAA-3′ and reverse 5′-GCTTCTTCCGACCACTCTTT-3′.

### MicroRNA extraction and purification:

MicroRNA was isolated using miRNAEasy Mini Kit (Qiagen, Germany) following the suppliers’ protocol. Mature miRNA was reverse transcribed to cDNA with QuantiTect Reverse Transcription (Qiagen, Germany). The expression level of miRNA-135b was identified by Quantitect SYBR Green PCR kit (Qiagen, Germany) using Veriti Thermal Cycler (Applied Biosystems, USA). miRNA-135b expression level was assessed by qPCR with the following primers: 5′-CGAGGCATACCGAAAA GT-3′ (Forward) and 5′-CCAGTGCAGGGTCCG AGGTA-3′ Reverse).

### Statistical analysis:

Statistical analyses were done using SPSS 23. The distribution of data was evaluated from scatter plots and histogram, supported by Shapiro Wilk test for normality. All the data were normally distributed. The difference of mi-RNA 135b expression and HOXA-10 mRNA expression between the observation group and the control group was analyzed using Student’s T-test. Meanwhile, Pearson’s correlation coefficient was performed to detect the correlation between the expression of miRNA-135b and mRNA expression of HOXA-10 and p<0.05 suggested that the difference was statistically significant.

## Results

Twenty three women who met the study requirements were divided into infertile group and fertile group as the study controls. The characteristics of the subjects, such as age, body mass index (BMI), menstrual cycle and endometrial thickness are provided in table 1. There were no significant differences in these variables between the two groups.

**Table 1.** Baseline conditions of subjects in infertility group and control group

	**Control (n=9)**	**Infertility (n=14)**
**Age (years) ^a^**	32.6±3.9	32.0±4.0
**BMI (*kg/m*^2^) ^a^**	24±0.78	24±0.87
**Menstrual cycle (d) ^a^**	28.9±0.95	28.7±0.97
**Endometrial thickness (*mm*) ^a^**	10.9±0.86	10.7±0.83

a: Data is normally distributed therefore value is presented in mean and standard deviation

**Figure 1.**
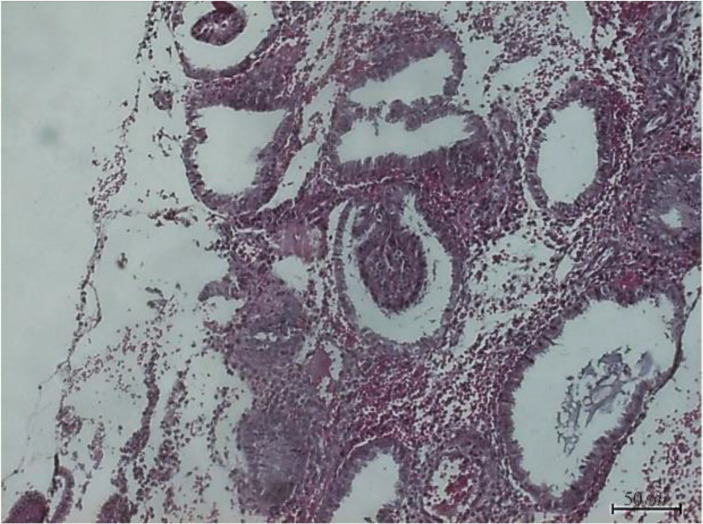
Representative samples of H&E-stained in the secretory endometrium histology

Endometrial biopsies were done during the secretory phase of the uterus by an experienced histopathologist followed by haematoxylin-eosin-stained (H&E) endometrial sections (Figure 1). Histology imaging was used to validate tissue collection during the secretory phase.

**Figure 2.**
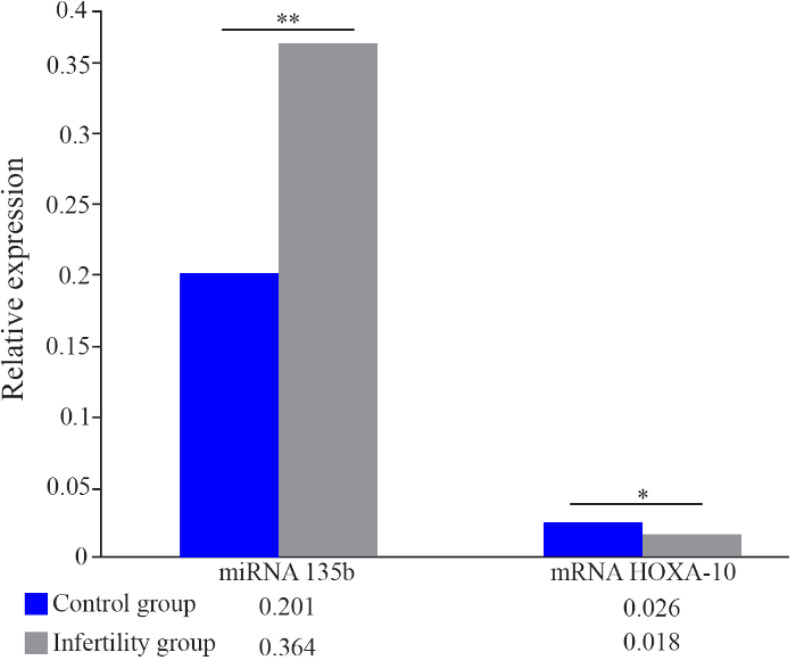
Relative expression of miRNA 135b and mRNA HOXA-10. The y-axis represents fold change in expression as determined by quantitative real-time PCR and is expressed as mean±SEM. Asterisk represents statistically significant difference between groups (*p<0.05, **p<0.01)

The expression of miRNA-135b and HOXA-10 mRNA isolated from the endometrial samples were measured through quantitative real-time PCR. HOXA-10 mRNA expression was lower by 0.69-fold (p=0.047) in the infertile group compared to the controls. On the other hand, the expression of miRNA-135b was significantly higher by 1.81-fold (p<0.01) in the endometrium samples from women diagnosed with infertility issues (Figure 2).

Pearson’s correlation coefficient on the expression of miRNA-135b and mRNA HOXA-10 mRNA in endometrium samples of the infertile group revealed a significant negative correlation between the two variables (p=0.021; r=−0.607). On the other hand, there was no correlation between these variables in the control group (p= 0.747; r=0.126).

## Discussion

The causes of repeated implantation failure (RIF) remain as unsolved complications that need to be addressed in order to improve the outcomes of ART (12). Endometrial receptivity, being one of the physiological aspects that are affected in RIF, is defined as the ability of the uterus to receive embryos thus permitting a successful pregnancy. The decrease in endometrial receptivity during a stimulated IVF cycle is proportional to the decrease in implantation rates.

MicroRNAs are responsible for regulating various genes, resulting in altered cellular processes and behaviors (13). Several previous studies have reported that miRNAs have contributed to implantation failure in infertile women by modifying the expression of genes associated with the efficiency of embryonic implantation in the receptive endometrium (14, 15). Alterations in endometrial mi-RNA expression could therefore serve as a useful marker to assess the endometrial receptivity in RIF cases so that the appropriate alternative therapy could be subjected to the patients.

This study utilized qPCR to evaluate the differential miRNA expressions in endometrial tissues of women with and without complications of infertility. Using this approach, potential role of mi-RNA-135b was discovered in regulating HOXA10 gene as a key factor in embryonic implantation.

Differential miRNA expression is apparent between the secretory phase and proliferative phase of the mensruation, which justifies the relationship between hormonal and cyclical variations and miRNA expression (16). Embryonic implantation involves various genes that partake in the signal transduction pathways and the cyclical variation of miRNA expression provides an opportunity to identify aberrant modifications in miRNA expression during the window of implantation when the endometrium is supposedly ready to receive the blastocyst. In the present investigation, levels of miRNA-135b were significantly higher in the endometrial secretory phase of the infertile group compared to the control group. A similar study conducted by Kresowik et al. reported that the expression of miRNA-135b was underexpressed in endometrial tissue obtained during the mid-secretory phase as compared to the proliferative phase (16).

The expression of HOXA10 gene, on the other hand, rises significantly during the mid-luteal phase and remains high from the time of implantation until the end of the reproductive cycle (13). Our data indicates that HOXA10 mRNA expression was 0.69-fold lower in eutopic endometrial tissues of women with infertility compared to those of the control subjects. Alteration in HOXA10 expression has been identified in several gynaecological disorders such as endometriosis, polycystic ovary syndrome, and other conditions related to abnormal implantation (17, 18). Therefore, HOXA10 gene expression in mid-secretory endometrium has been reported as a receptivity marker that could assist the diagnosis of implantation impairment in infertile women.

Since miRNA is known to regulate many genes or pathways, identification of common target genes is necessary to understand the molecular mechanism of implantation failure in infertile patients (2). Based on our results, the increase in the levels of miRNA-135b had caused the decrease in HOXA10 mRNA expression in endometrial cells of the infertile women. In accordance with our study, Petracco et al. also reported that higher expression of miRNA 135b led to the decrease of HOXA10 expression in endometrial cells of endometriosis patients (11).

## Conclusion

In conclusion, our result seems to suggest that increased expression of miRNA-135b may down-regulate endometrial receptivity and might suppress genes required for implantation. HOXA-10 gene expression was decreased in the endometrium of infertile women which is likely caused by the increased miRNA 135b expression. Furthermore, miRNA analysis has opened a new and intriguing way of understanding the pathogenesis of repeated implantation failure in women with infertility.
